# Supplementary material for: Functional basis of electron transport within photosynthetic complex I
Source: Nat Commun. 2021 Sep 10;12:5387. doi: 10.1038/s41467-021-25527-1 (PMC8433477; doi:10.1038/s41467-021-25527-1)
Supplement: Supplementary file 1 — Supplementary Information [file 41467_2021_25527_MOESM1_ESM.pdf]

Supplementary Information

Functional basis of electron transport within photosynthetic complex I

Katherine H. Richardson[1,2], John J. Wright[1,3], Mantas Šimėnas[4], Jacqueline Thiemann[5], Ana M. Esteves[1], Gemma McGuire[1,2], William K. Myers[6], John J.L. Morton[4,7], Michael Hippler[8,9], Marc M. Nowaczyk[5], Guy T. Hanke[1*], Maxie M. Roessler[2*]

[1]School of Biological and Chemical Sciences, Queen Mary University of London, Mile End Road, London E1 4NS, UK

[2] Department of Chemistry, Imperial College London, Molecular Sciences Research Hub, London W12 0BZ, UK

[3]Medical Research Council Mitochondrial Biology Unit, Wellcome Trust/MRC Building, Hills Road, Cambridge CB2 0XY, UK

[4]London Centre for Nanotechnology, University College London, London WC1H 0AH, UK

[5]Plant Biochemistry, Faculty of Biology and Biotechnology, Ruhr University Bochum, 44780 Bochum, Germany

[6]Inorganic Chemistry, University of Oxford, South Parks Road, OX1 3QR Oxford, UK

[7]Department of Electronic & Electrical Engineering, UCL, London WC1E 7JE, UK

[8]Institute of Plant Biology and Biotechnology, University of Münster, 48143 Münster, Germany

[9]Institute of Plant Science and Resources, Okayama University, Kurashiki, Japan

*Correspondence to: m.roessler@imperial.ac.uk and g.hanke@qmul.ac.uk

Contents

Functional basis of electron transport within photosynthetic complex I 1
Supplementary Note 1. Assignment of the [4Fe–4S] clusters of PS-CI using relaxation filtered EPR . 3
Supplementary Note 2. Simulation of DEER spectra and analysis 5
Simulation of orientation selective DEER to structurally assign the FeS clusters of PS-CI 5
Comparison with respiratory complex I 7
Supplementary Tables 8
Supplementary Table 1. Tandem mass spectrometry of *T. elongatus* PS-CI. 8
Supplementary Table 2. Tandem mass spectrometry of *Synechocystis* PS-CI. 9
Supplementary Table 3. Summary of FeS cluster *g* values and *g* strain in PS-CI 10
Supplementary Table 4. FeS cluster *g* values in R-CI from different species. 10
Supplementary Table 5. Experimental DEER parameters for the positions in Supplementary Fig. 3 and DEER traces in Supplementary Fig. 4. 11
Supplementary Figures 12
Supplementary Fig 1. Relaxation filtering of the [4Fe – 4S] clusters of *T. elongatus* PS-CI 12
Supplementary Fig 2. Relaxation filtering of the [4Fe – 4S] clusters of *Synechocystis* PS-CI 13
Supplementary Fig 3. DEER pump and detection pulse set up 14
Supplementary Fig 4. Complete set of experimental DEER spectra of PS-CI and simulations for best fit *g* tensors and two different sets of SPFs 15
Supplementary Fig 5. Complete set of experimental DEER spectra of PS-CI with fits resulting from lowest residual simulations 16
Supplementary Fig 6. Orientation selective DEER simulation parameters for two [4Fe-4S] clusters 17
Supplementary Fig 7. EPR-based Potentiometric Redox Titrations 18
Supplementary Fig 8. Lack of N0 signal in *T. elongatus* PS-CI at −431 mV 19
Supplementary Fig 9. Synthetic DNA sequence from *Synechocystis* used to introduce a His-tag to NDH-J 20
References 21

Supplementary Note 1. Assignment of the [4Fe–4S] clusters of PS-CI using relaxation filtered EPR

The local environment of a paramagnetic species, and hence the extent of spin-orbit coupling of excited states into the ground state, causes deviation from the $g = 2.0023$ EPR signal of a free unpaired electron. Protein [4Fe-4S] clusters typically display axial or rhombic EPR signals with g values around 2.01 and 1.94 (where g values less than 2.0023 are the result of antiferromagnetic coupling between Fe centres). The exact g values, conformational strain and symmetry vary for each cluster, reflecting its particular environment. Clusters in similar structural environments have similar g values (Supplementary Table 4). PS-CI contains three [4Fe-4S] clusters which all contribute to some extent to the fully reduced spectra (Figure 2). The N2 cluster is structurally almost identical to R-CI (Figure 1) and therefore the g values are expected to be extremely similar in PS-CI. This is indeed the case (see Supplementary Table 2 & 3). The CW EPR spectra contain two fully reduced clusters, N2 and a second, defined here as N1 (note that N1a and N1b are [2Fe-2S] clusters in respiratory complex I). N0 is the third semi-reduced cluster that exhibits a very broad EPR signal (Figure 2). The three FeS cluster EPR signals overlap, making accurate assignment of their g values from CW EPR alone difficult.

As well as g values and strain, paramagnetic species have a spin-lattice relaxation time (T_1) and spin-spin relaxations time (T_2) influenced by their environment. Pulsed EPR was used to vary the contribution of different clusters to the EPR spectrum based on their relaxation times, by altering parameters of the applied microwave pulse sequence. The differing relaxation times can be used comparatively to filter the contribution of centres with distinct relaxation properties. Such 'relaxation-filtered' spectra were used to deduce the g values and strain for the three overlapping clusters, i.e. finding the values that fit each [4Fe-4S] cluster consistently (Supplementary Figures 1 & 2). The [4Fe-4S] clusters overlap greatly and do not have significantly different relaxation times. Therefore orientation-dependent relaxation could not be accounted for. Instead, the g strain was allowed to vary in individual fits and the best-fit for CW spectra and EDFS without T_1 filtering was used for the final fits (Supplementary Figures 1 and 2).

The first pulsed technique used to disentangle the FeS EPR signals in PS-CI was T_1-relaxation filtered EDFS[1]. In this experiment, an initial π pulse inverts the spin magnetisation followed by a Hahn echo detection sequence applied after the filtering time T_f. By varying T_f, spin species having substantially different T_1 relaxation time can be resolved. PS-CI recovers N2 and N0 at long T_f in both species (Supplementary Figure 1a & 2a). This indicates both N2 and N0 are recovered at relatively long T_1 (~20 and 19 µs respectively). N1 is recovered at intermediate T_f indicating its T_1 is short compared to the other clusters (~4 µs). T_1 relaxation times were calculated based on the limited number of T_f times used in Supplementary Figure 1a & 2a, therefore are estimates only.

Different measurement temperatures were also used to disentangle the overlapping spin species, which have different relaxation times. If a species is slow relaxing, it will begin to saturate at higher temperatures than faster relaxing species. As we decrease the temperature, the relative intensity of N1 and N0 increases compared to N2 for PS-CI from both species (Supplementary Figure 1b & S2b). N2 and N0 are also visible at higher temperatures than N1. This indicates N2 > N0 > N1 in T_1 relaxation time (i.e. N1 is the fastest relaxing cluster).

The above trend in T_1 relaxation time is further corroborated by the spin magnetization recovery, which depends on the shot repetition time (SRT) of the pulse EPR experiment. Slow relaxing species are not observed at short SRT due to saturation. The EPR signal intensity of N2 in *T. elongatus* PS-CI is

reduced relative to N1 and N0 with decreasing SRT, therefore N2 is the slowest relaxing (Supplementary Figure 1c). N2 also decreases with shorter SRT in *Synechocystis* PS-CI, however the effect is much less pronounced (Supplementary Figure 2c).

Any species which has lost coherence during the spin evolution time τ will not be detected in an EDFS. By varying τ, EPR FeS cluster signals are filtered primarily based on their spin-spin relaxation time T_2. Signal N2 has a relatively long T_2 of ~1.4 µs, similar to that observed for single FeS clusters[2]. It is therefore the slowest relaxing in both species, consistent with its slow relaxing T_1 (Supplementary Figure 1d & 2d). N0 is filtered at long τ indicating that its T_2 is relatively short (~0.55 µs). The relatively long T_1 and short T_2 for N0 may be due to its proximity (~11 Å) to a fully reduced cluster (N1) or be an effect of inorganic ions present in the buffer given that N0 is almost solvent-exposed (based on the DEER assignment). N1 appears to also have a fast relaxation time (T_1 = 4 µs and T_2 = 0.5 µs) in both species (Supplementary Figure 1d & 2d), consistent with its assignment of being the middle cluster, sandwiched between two paramagnets.

The differences in signal contribution afforded by varying these T_1 and T_2-dependent parameters enables a robust model of the *g* values and strains to be built for each induvial cluster (see Supplementary Table 3). The parameters thus determined yield good fits of all pulsed and CW spectra presented in this work. In the case of PS-CI, N2 has the slowest relaxation times; N1 has relatively fast relaxation times; and N0 has an unusual combination of slow T_1 with a fast T_2. To reflect the assignment of the EPR signals to their structurally defined clusters and to avoid confusion between the R-CI [4Fe-4S] clusters (N2, N3, N4 and N5) or [2Fe-2S] clusters (N1a or N1b), we have assigned the nomenclature N2, N1 and N0 to PS-CI.

Supplementary Note 2. Simulation of DEER spectra and analysis

An in-house programme that enables the simulation of orientation selective DEER spectra with highly delocalised spin centres, adapted from a previously reported programme[3], was used to simulate the DEER spectra. Briefly (further details below), all possible orientation selective DEER spectra were calculated for each possible assignment model and the resulting simulated spectra were compared with experimental data. The simulations take into account the experimental conditions used, the FeS cluster properties (cyroEM structure to obtain distances, *g* values and strain) and a local spin model for the spin Hamiltonian of [4Fe – 4S] clusters with spin projection factors $k_{1,2}$ =+1.17 $k_{3,4}$ =-0.67. Best fits to the experimental data were selected based on least-square fit residuals.

Simulation of orientation selective DEER to structurally assign the FeS clusters of PS-CI

Full accounts of the simulation algorithm[4], local spin model theory[5,6] and application to FeS cluster containing proteins[3] have been presented previously. The spin Hamiltonian for a pair of clusters (A and B) is written in terms of the intracluster spin-spin coupling interactions H_{int}, the Zeeman interaction H_z, the exchange interaction H_{ex}, and the dipolar interaction H_{dd}:

$$H = H_{int} + H_z + H_{ex} + H_{dd} \qquad \text{Eq. 1}$$

The intracluster Hamiltonian (H_{int}) describes the interaction between the Fe spins, which couple to give an effective spin for the cluster; in clusters discussed here S = 1/2 is the ground state. The local spin model describes the individual spins of each Fe using the Wigner–Eckart theorem, where the spin column vector operator (**S**) for the *i*-th Fe in the cluster in cluster A (or B) is expressed in terms of the spin projection factor k_i:

$$\mathbf{S}_{Ai} = k_i \mathbf{S}_A. \qquad \text{Eq. 2}$$

The Zeeman interaction (H_z) is calculated from the measured **g** matrices for the clusters, resulting from the weighted sum of the **g**-matrix contributions of each Fe center:

$$\mathbf{g_A} = \sum_i k_i \mathbf{g} \mathbf{A_i}. \qquad \text{Eq. 3}$$

The exchange interaction (H_{ex}) is set to zero in our analysis as it is expected to be very small when the distance between the paramagnetic centres is >10 Å.

The dipolar frequencies observed in the DEER experiment result from the dipolar Hamiltonian:

$$H_{dd} = \frac{\mu_0 \beta^2}{4\pi} \sum_i \sum_j \boldsymbol{S}_{A_i}^T \frac{g_{A_i}^T g_{B_j} - 3\left(g_{A_i}^T \boldsymbol{n}_{ij}\right)\left(\boldsymbol{n}_{ij}^T g_{B_j}\right)}{r_{ij}^3} \boldsymbol{S}_{B_j}, \qquad \text{Eq. 4}$$

where **S** is the electron spin column vector operator, r_{ij} is the distance, $\boldsymbol{n}_{ij}$ is the unit column vector ($\boldsymbol{n}_{ij} = \boldsymbol{r}_{ij}/|\boldsymbol{r}_{ij}|$). The **g** matrices used in the DEER simulations were experimentally determined, equivalent to an average value over the Fe subsites in the cluster. The averaging error introduced by this can be considered to be incorporated into the spin projection factors. As the dipolar frequency at a particular orientation is proportional to the *g* value this will introduce a maximum error for N0 of $(g_{max} - g_{avg})/g_{avg}$ = 0.15/2 = 7.5%.

The dipolar coupling frequency, ω_{dd}, is approximated to[2,10]:

$$\omega_{dd}^m = \frac{\mu_0 \beta^2}{2h} g_A^m g_B^m \sum_i \sum_j k_{A_i} k_{B_j} \frac{3\cos^2 \psi_{ij}^m - 1}{r_{ij}^3}, \qquad \text{Eq. 5}$$

where g_A^m and g_B^m are the experimental *g* values observed at the orientation of the unit magnetic field vector $B_0^m(\theta,\phi)$ and ψ is the angle between $B_0^m(\theta,\phi)$ and n_{ij}, the unit vectors between two FeS cluster Fe subsites (see Supplementary Figure 6). The subscript *m* refers to a particular (θ,ϕ) value for the unit magnetic field vector in the molecular frame. The simulation program samples $B_0^m(\theta,\phi)$ over half a unit sphere to include all possible orientations (as the spin Hamiltonian has inversion symmetry in the general case).

At X-band, FeS EPR spectra are significantly broader than microwave pulse excitation bandwidth, therefore only a subset of orientations, with respect to $B_0^m(\theta,\phi)$ will be excited by a microwave pulse. The DEER time trace contribution for orientation $B_0^m(\theta,\phi)$, where f_A^m of the A-spin (observed spins) spectrum at this orientation is excited is given by:

$$y_{\mathrm{sim}}^{\mathrm{DEER},m}(t) = f_A^m\{(1-c_{\mathrm{mod}}^m) + c_{\mathrm{mod}}^m \cos(\omega_{\mathrm{dd}}^m t)\}. \qquad \text{Eq. 6}$$

If f_A^m = 0, the orientation will not contribute to the simulation. If it is not, the intensity of *m*-th orientation with dipolar frequency ω_{dd} weighted by c_{mod}^m and the nonmodulated part by $1-c_{\mathrm{mod}}^m$. The modulation parameter, c_{mod}^m, depends on the fraction B-spin resonance at $B_0^m(\theta,\phi)$ orientation that is excited by the pump pulse. Both these quantities are calculated by the integral between the A-spin (B-spin) spectrum at the orientation $B_0^m(\theta,\phi)$ and the position of the detection (pump) pulse profile.

The DEER traces are simulated for the experimental pump and detection microwave pulse positions (and relevant parameters) using the spin projection factors (SPF) for ferredoxin-type FeS clusters (here $k_{1,2}$ = +1.17 $k_{3,4}$ = −0.67 or $k_{1,2}$ = +1.35 $k_{3,4}$ = −0.85) that were previously found to be the suitable for R-CI[3], experimentally determined *g* values, strain, and cluster coordinates from the published cryo-EM structure (PDB 6HUM)[9]. Simulations were generated for *g* tensors through the FeS cluster faces (6 possible combinations for a rhombic cluster), or along the Fe-Fe vectors (6 possible combinations for a rhombic cluster), for all possible assignments of the clusters SPFs, resulting in 576 combinations for a given rhombic cluster pair. The simulations were weighted to the refocused echo intensity (measured with the DEER detection sequence) of the simulated detection spin ($A^{N\mathrm{X}}$, see Table S3), for model A with:

$$y_{sim}^{tot}(t) = A^{N2}y_{sim}^{N2}(t) + A^{N1}y_{sim}^{N1}(t) + A^{N0}, \qquad \text{Eq. 7}$$

and for model B with:

$$y_{sim}^{tot}(t) = A^{N2}y_{sim}^{N2}(t) + A^{N1} + A^{N0}y_{sim}^{N0}(t). \qquad \text{Eq. 8}$$

Simulations were based on one interacting cluster pair (at a centre-to-centre distance of 26 Å, see Figure 1), plus decay due to relaxation from the third cluster. No significant difference in the fits was observed when all three potential interactions were taken into account in the simulations as the middle cluster is <15 Å from the other two, therefore the modulation frequency is too high to be observed with the DEER pulse sequence employed. The best-fit models are selected based on the residuals between the experimental and simulated traces for each model. Model A did not produce a good fit for the lowest residual *g* tensor and SPF combination for all DEER traces (Supplementary Figure 4), either with $k_{1,2}$ = +1.17 $k_{3,4}$ = −0.67 or with $k_{1,2}$ = +1.35 $k_{3,4}$ = −0.85, which are both accepted value for ferredoxin-type Fe-S clusters are present in PS-CI. A reasonable fit with Model A could not even be obtained when *g* tensors and SPFs were allowed to vary between traces, although this is physically meaningless (Supplementary Figure 5). The top 10% of fits were visually inspected to ensure the best fit was not a local minimum in the residual fits. None of the top 10% of fits for model A

produced a better fit than those presented in Supplementary Figure 4 & 5. The top 2% of fits for model B produced reasonable fits and the best was selected by visual inspection.

The modulation depth calculated for the spin pairs for model B matched well with the experimentally observed depth of ~0.08. The *g* tensors of the clusters that are the main contributors to the observed DEER spectra (N0 and N2, Model B) were found to be very similar to the R-CI N2 and N4 tensors reported previously[3].

Comparison with respiratory complex I

The experimentally observed modulation frequencies in the DEER spectra are similar to those previously reported for R-CI[3]. The simulations used to model the respiratory vs photosynthetic complex differ in two respects.

The first is the structure of N0 compared to N4 (both at ca. 26 Å from N2), as the cluster coordinates and principal *g* values are different (Table S3 & S4) (whereas those of the respective N2 clusters are very similar). The overall cluster to cluster distances (taking centre-to-centre distances) only vary ~0.2 Å and hence does not have a profound effect on the modulation frequency. As the FeS cluster EPR signals are highly anisotropic, and the signals overlap, the extent to which principal *g* values affect modulation of the DEER spectra depends on the pump and probe positions chosen and can be very minimal. The good fit for both models A and B at DEER position 1 (Supplementary Figure 4) exemplifies the importance of multiple pump and probe position to assign orientation selective DEER spectra. Hence, although the *g* values of N4 and N0 differ slightly, their similar relative orientation (and hence distance) gives rise to similar orientation selective DEER spectra (for which pump/probe positions are comparable).

The second difference in the models is the calculation of modulation depth. In both cases simulated spectra are weighted to the signal intensity from individual clusters at different probe positions (as determined from EDFS, Figure 2b). In R-CI the cluster adjacent to N2 is not reduced, and therefore does not contribute to the DEER spectra. N4, N3 and N1b (a [2Fe 2S] cluster) all could have potentially contributed, and different models were calculated to reflect this, with N4 at 26 Å from N2 providing the best fit to experimental data. PS-CI contains only three FeS clusters which are all reduced to some extent. Therefore, all models were calculated using weights for both the clusters involved in the contributing distances. The accuracy of the simulated modulation depth and decay compared to experimental data, using the model for a partially reduced cluster N0, model B, confirms that the assignment is correct.

Supplementary Tables

Supplementary Table 1. Tandem mass spectrometry of *T. elongatus* PS-CI.

Subunit	ID	kDa	Protein score	Coverage (%)
NdhA	Q8DL32	41.3	25.44	22.96
NdhB	Q8DMR6	55.1	20.15	8.74
NdhC	Q8DJ02	13.7		
NdhD	Q8DKY0	56	138.31	21.21
NdhE	Q8DL29	11.2	18.32	43.56
NdhF	Q8DKX9	71.9	0.00	6.25
NdhG	Q8DL30	21.6	117.93	25.50
NdhH	Q8DJD9	45.2	344.24	63.20
NdhI	Q8DL31	22.4	72.55	47.96
NdhJ	Q8DJ01	19.2	66.88	64.29
NdhK	Q8DKZ4	25.7	145.81	35.02
NdhL	Q8DKZ3	11.6		
NdhM	Q8DLN5	12.6	444.83	49.55
NdhN	Q8DJU2	8.3	68.95	79.33
NdhO	Q8DMU4	7.86	110.44	78.57
NdhP		4.9	0.00	36.36
NdhQ		4.7		
NdhS	Q8DL61	8.1	80.61	62.16
NdhV		13.6	17.50	55.20

Supplementary Table 2. Tandem mass spectrometry of *Synechocystis* PS-CI.

Subunit	ID	kDa	Protein score	Coverage (%)
NdhA	P26522	40.5	55.40	24.5
NdhB	P72714	55.4	38.43	15
NdhC	P19045	13.7		
NdhD	P32421	57.5	113.01	18.5
NdhE	P26524	11.5	16.95	13.6
NdhF	Q55429	74.4	21.98	9.8
NdhG	P26523	21.5	41.19	12.1
NdhH	P27724	45.5	252.8	72.1
NdhI	P36525	22.2	116.47	65.8
NdhJ	P19125	20.6	69.58	41.3
NdhK	P19050	27.3	112.69	81.5
NdhL	P27372	9.3	2.72	12.5
NdhM	P74338	14.1	59.75	57.9
NdhN	P74069	17.6	115.16	72.7
NdhO	P74771	8.3	38.51	63.9
NdhP				
NdhQ				
NdhS	P74795	6.6	23.63	65.5
NdhV				

Supplementary Table 3. Summary of FeS cluster *g* values and *g* strain in PS-CI. See Supplementary note 2 for details of the assignment. [a] Values taken from[9]. Additional simulation parameters for CW spectra (Fig. 2a) and echo-detected field sweeps (Fig. 2b). Relative weightings from EDFS for *T. elongatus* used in DEER trace simulations (Fig 2c & d, Supplementary Figure 4 & 5 and Equations 7 and 8). Estimated relaxation times at 10K for each cluster based on Supplementary Figure 1 & 2.

		T. elongatus [a]	***T. elongatus***		***Synechocystis***		***T. elongatus***		
		g value	*g* value	*g* strain	*g* value	*g* strain	**Relative weighing (A)**	**T_1 relaxation time (µs)**	**T_2 relaxation time (µs)**
N2	g_x	1.922	1.922	0.0092	1.921	0.0102	0.3972	20	1.4
	g_y	1.922	1.922	0.0112	1.921	0.0102			
	g_z	2.055	2.055	0.0087	2.055	0.0064			
N1	g_x	1.899	1.907	0.0143	1.884	0.0204	0.3908	4	0.5
	g_y	1.911	1.913	0.0169	1.918	0.0158			
	g_z	2.045	2.045	0.0087	2.047	0.0200			
N0	g_x	1.854	1.852	0.0376	1.851	0.0231	0.2120	19	0.55
	g_y	1.914	1.899	0.0201	1.867	0.0261			
	g_z	2.041	2.064	0.0271	2.07	0.0272			

Supplementary Table 4. FeS cluster *g* values in R-CI from different species.

		B. taurus[3]	***Y. lipolytica***[10]	***E. coli***[11]	***T. thermophilus***[12]
N2	g_x	1.921	1.925	1.903	1.93
	g_y	1.927	1.929	1.903	1.94
	g_z	2.054	2.053	2.05	2.048
N4	g_x	1.886	1.894	1.894	1.801
	g_y	1.928	1.939	1.94	1.949
	g_z	2.107	2.103	2.088	2.063

Supplementary Table 5. Experimental DEER parameters for the positions in Supplementary Fig. 3 and DEER traces in Supplementary Fig. 4.

Position	Magnetic field (mT)	Detection frequency ω_A (GHz)	Pump frequency ω_B (GHz)	Detection pulse π_A (ns)	Pump pulse π_B (ns)
1	338.9	9.116	9.676	20	14
2	352.6	9.484	9.316	24	12
2*	352.4	9.484	9.316	24	10
3	346.0	9.350	9.616	24	16
4	344.1	9.256	9.574	24	12
5	344.1	9.256	9.665	24	14
6	350.8	9.512	9.359	12	20
7	357.4	9.588	9.361	20	18
8	356.9	9.360	9.590	16	18
9	357.0	9.302	9.590	16	18

Supplementary Figures

Supplementary Fig 1. Relaxation filtering of the [4Fe – 4S] clusters of *T. elongatus* PS-CI. Unless otherwise stated measurements were performed at 10 K, τ = 0.25 µs, π = 32 ns, shot repetition time (SRT) = 2.04 µs. **a.** T_1-filtered (π -T_f-π/2–τ–π–τ–echo) spectra of *T. elongatus* PS-CI, filtration times (T_f, µs) indicated on the left **b.** Temperature dependence, **c.** shot repetition time (SRT, µs) dependence (7K), and **d.** τ dependence (µs) of the Hahn echo (π/2–τ–π–τ–echo) of the FeS clusters signals of *T. elongatus* PS-CI. Raw data in black, sum of simulations in red. Percentage contributions of N2 in pink, N1 in purple and N0 in grey. See Supplementary Note 2 for more details.

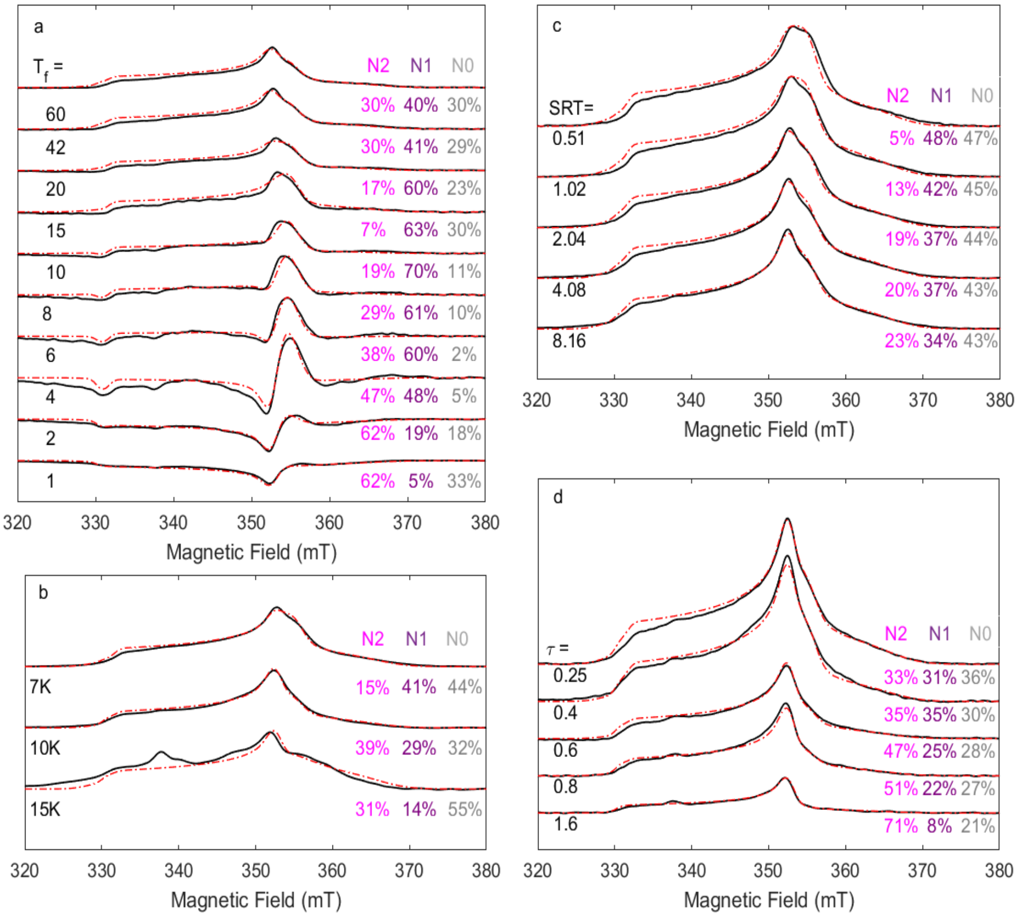

Supplementary Fig 2. Relaxation filtering of the [4Fe – 4S] clusters of *Synechocystis* PS-CI. Unless otherwise stated measurements were performed at 10K, τ = 0.25 μs, π = 32 ns, SRT = 8.16 μs. **a.** T_1-filtered (π -T_f- π/2–τ– π –τ–echo) spectra of *Synechocystis* PS-CI at, filtration times indicated on the left (T_f, μs) **b.** Temperature dependence, **c.** shot repetition time (SRT, μs) dependence, and **d.** τ dependence (μs) of the Hahn echo (π/2–τ–π–τ–echo) of the FeS clusters signals of *Synechocystis* PS-CI. Raw data in black, sum of simulations in red. Percentage contribution of N2 in pink, N1 in purple and N0 in grey. The peak observed at about 345 mT in the *Synechocystis* spectra is a g = 2 signal originating from an organic radical. See Supplementary Note 2 for more details.

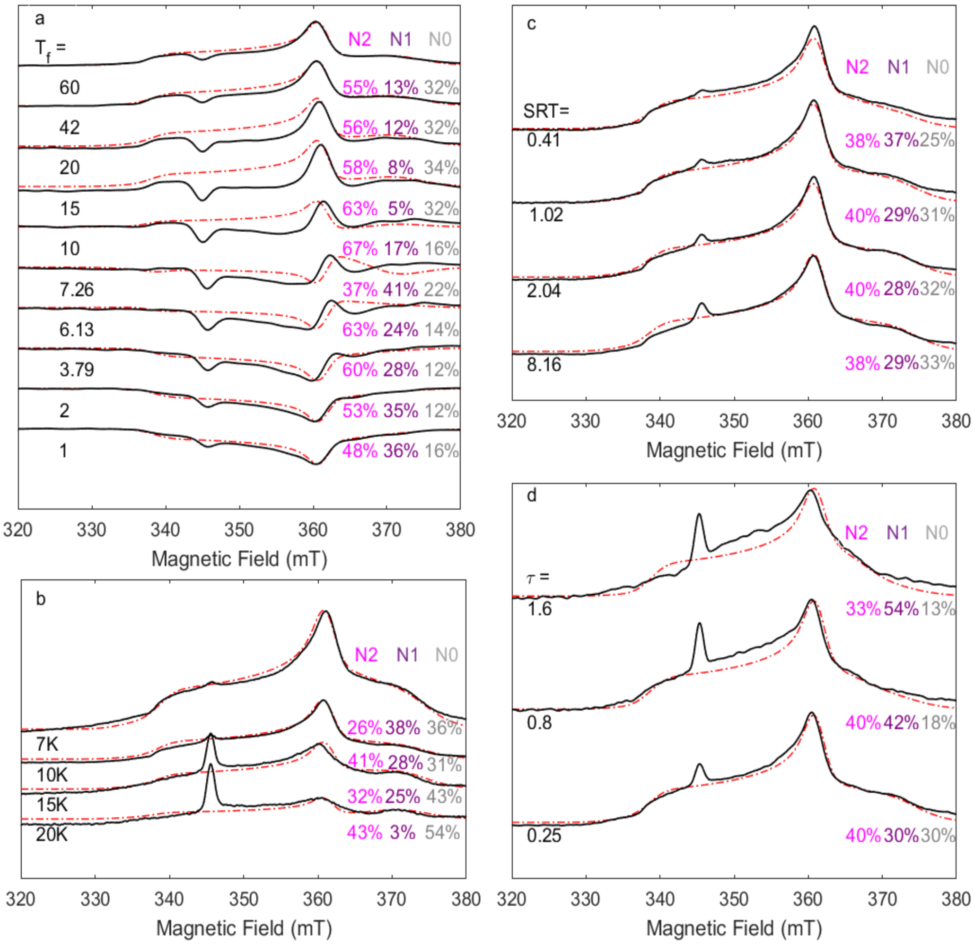

Supplementary Fig 3. DEER pump and detection pulse set up. **a.** Representation of the orientation selection of the microwave pulses for position 2. The pump pulse excitation profile for a 12 ns π-pulse at frequency ω_B = 9.316 GHz (red) and the detection pulse excitation profile for a 24 ns $\pi/2$-pulse at ω_A = 9.484 GHz (black) applied to *T. elongatus* PS-CI. Only a fraction of each spectrum is excited by the microwave pulses. The fixed field position of the DEER measurement is at 352.6 mT. **b.** Pump pulse positions in red, detection pulse positions in black corresponding DEER traces in Supplementary Fig. 4 (EDFS 10K, τ = 0.134 µs, π = 24 ns, SRT = 2.04 µs). Full experimental parameters in Supplementary Table 5, raw data in black, sum of simulations in red, N2 in pink, N1 in purple, N0 in grey. Simulation used the *g* values and strains in Supplementary Table 3 with N2:N1:N0 ratios 1.00:0.92:0.90. See Supplementary Table S3 for simulation parameters for the echo-detected field sweep.

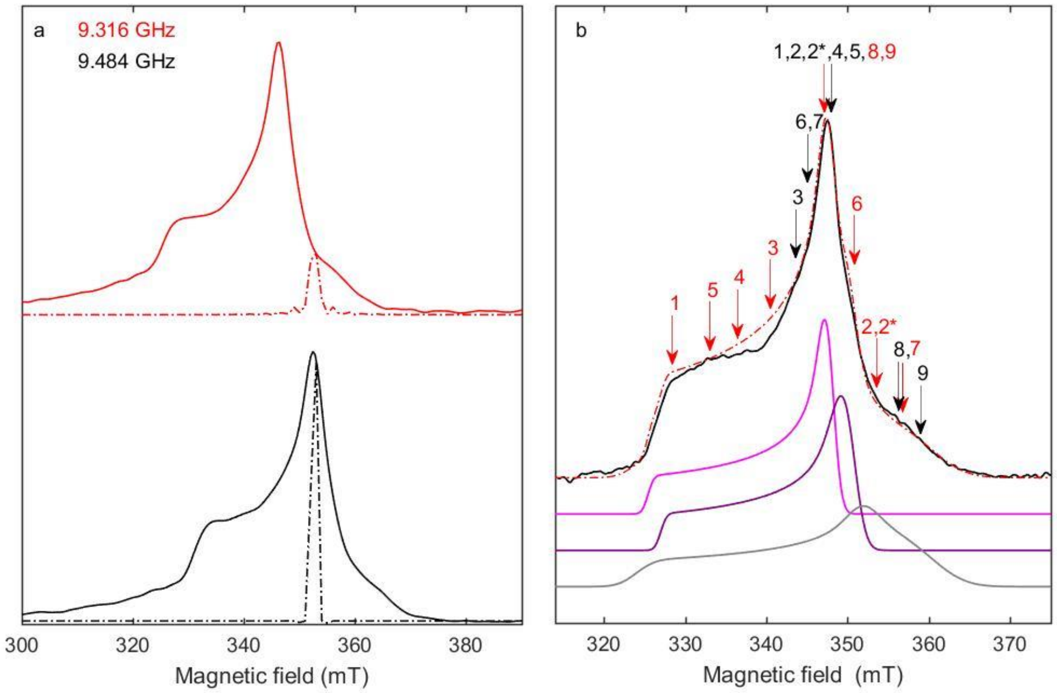

Supplementary Fig 4. Complete set of experimental DEER spectra of PS-CI and simulations for best fit *g* tensors and two different sets of SPFs. **a.** Model A in which N2 and N1 are 26 Å apart; SPFs: $k_{1,2}$ = +1.17 $k_{3,4}$ = −0.67. **b.** Model B in which N2 and N0 are 26 Å; SPFs: $k_{1,2}$ = +1.17 $k_{3,4}$ = −0.67. **c.** Model A in which N2 and N1 are 26 Å apart; SPFs: $k_{1,2}$ = +1.35 $k_{3,4}$ = −0.85. **d.** Model B in which N2 and N0 are 26 Å; SPFs: $k_{1,2}$ = +1.35 $k_{3,4}$ = −0.85. DEER spectra from *T. elongatus* measured at 10 K, magnetic field (mT), detection (ω_A, GHz) and pump pulse (ω_B, GHz) positions (corresponding to the pump and detection pulse positions shown in Supplementary Fig. 3), detection π length (π_A, ns) and pump π length (π_B, ns) listed in Supplementary Table 5; raw data in black. The models correspond to the cluster order indicated below, distances calculated from the intercluster coordinates of PDB:6HUM. All traces are normalised to the intensity at zero time. A linear baseline was subtracted from the traces to remove intermolecular decay. The modulation depth is indicated as a scale bar.

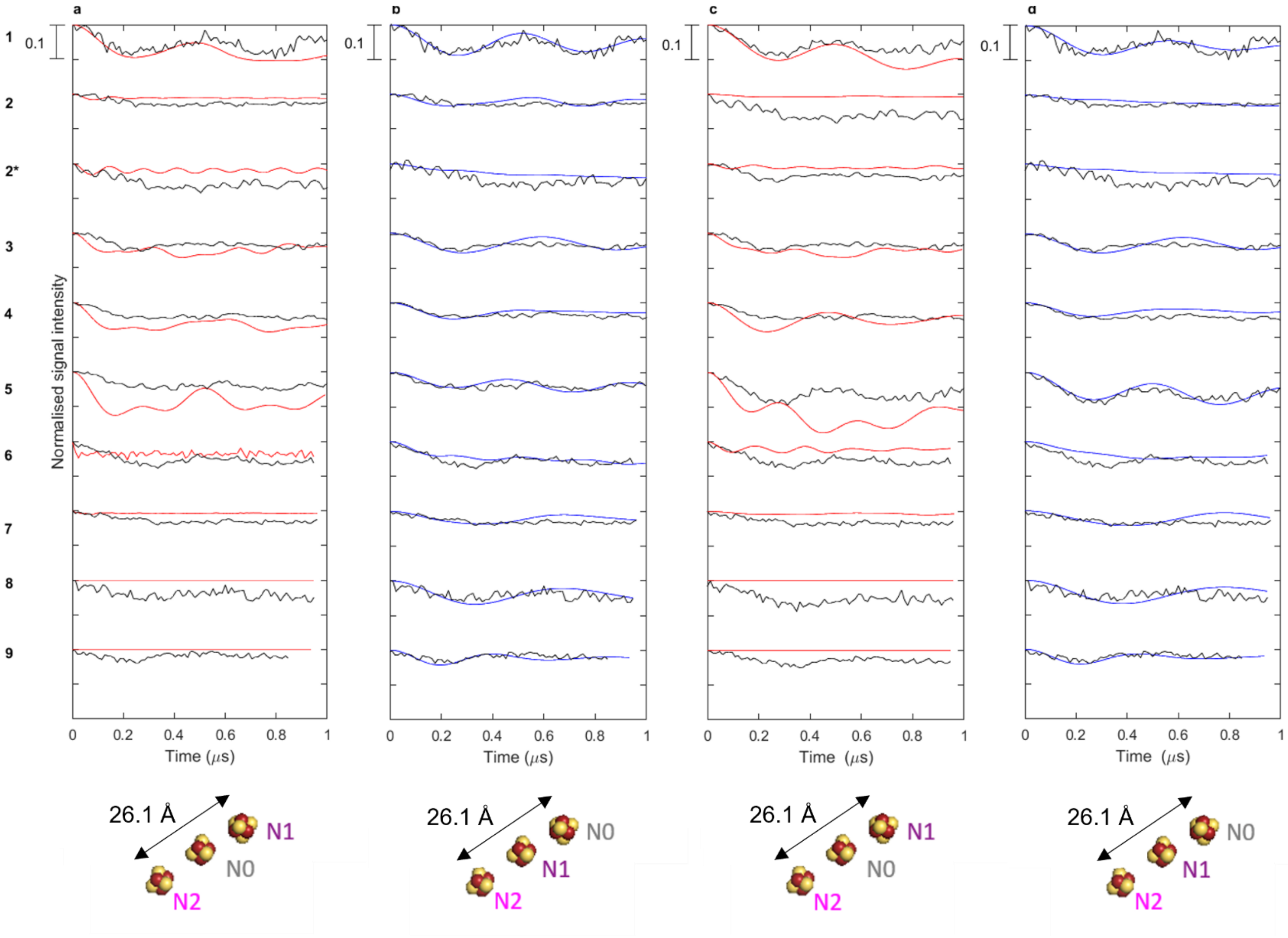

Supplementary Fig 5. Complete set of experimental DEER spectra of PS-CI as shown in Supplementary Fig 4, but with fits resulting from lowest residual simulations for each trace 1-9 when there are no structural constraints for $k_{1,2} = +1.17$ $k_{3,4} = -0.67$ (*g* tensor orientation and SPF assignment are allowed to vary from trace to trace). **a.** Model A in which N2 and N1 are 26 Å apart. **b.** Model B in which N2 and N0 are 26 Å apart. Note that this scenario is not physically meaningful but emphasises that Model A still does not result in any reasonable fit.

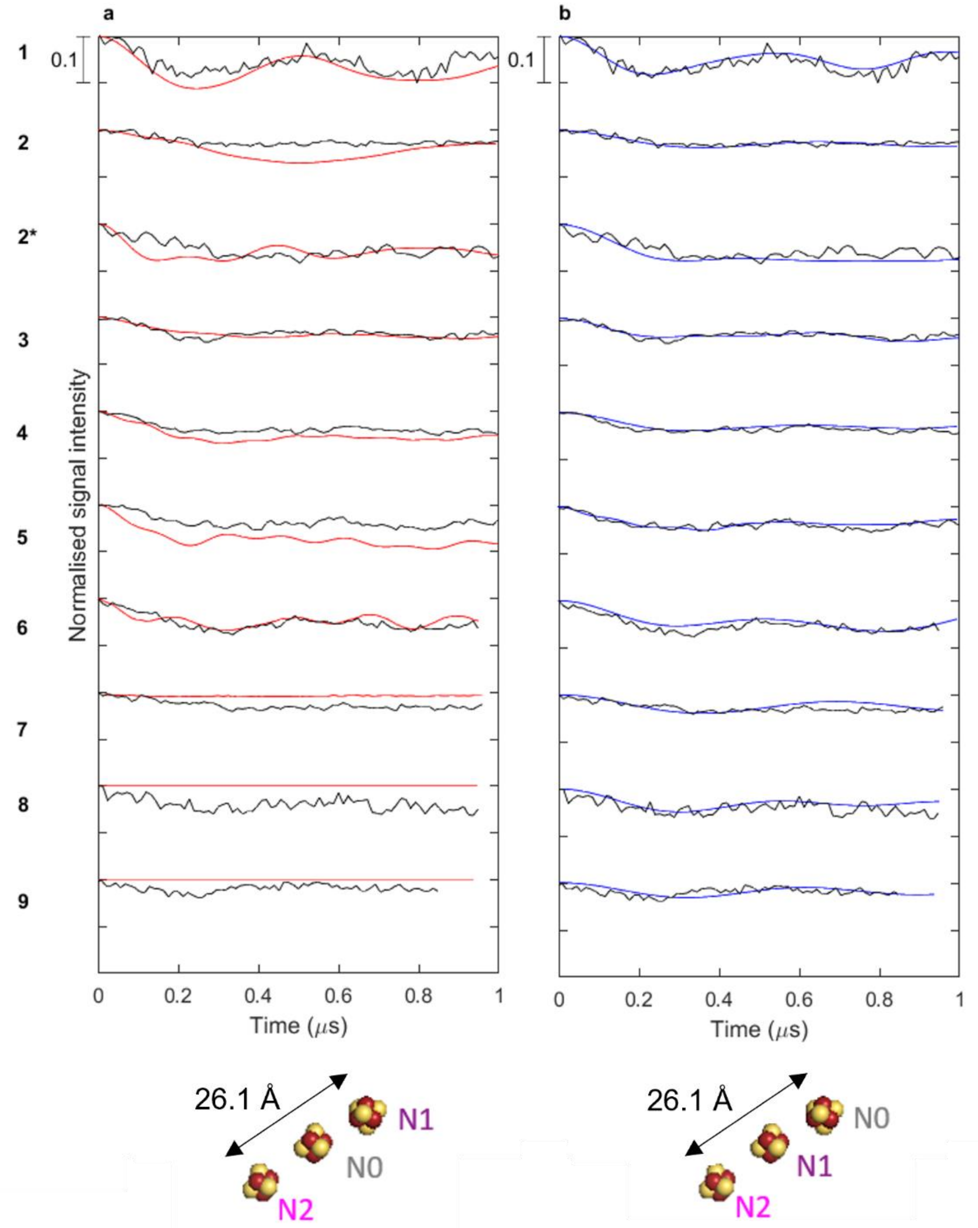

Supplementary Fig 6. Orientation selective DEER simulation parameters for two [4Fe-4S] clusters, with N2 and N0 as example. The unit magnetic field vector, $\mathbf{B}_0{}^m$, is defined by the angles Φ and θ. $\mathbf{n}_{ij}$ is the unit vector between the Fe ions i and j, r_{ij} is the distance between them. Ψ_{ij}^{m} is the angle between $\mathbf{B}_0{}^m$ and $\mathbf{n}_{ij}$. Schematic representation of the *g*-axis orientations in clusters N2 and N0 from the best-fit simulations for model B. The Fe subsites are shown in red, the S subsites in yellow. The cluster coordinates are taken from the structure of *T. elongatus* PS-CI (PDB 6HUM) and the (x,y,z) coordinates used in the structure PDB file define the molecular axes. The x, y, and z axes of the *g* matrices for N2 and N0, relative to the molecular axes, are: N2 x-axis = (-0.4760 , 0.5668 , -0.6724), y-axis = (-0.8209, -0.5606, 0.1086), z-axis = (-0.3154, 0.6037, 0.7322); N0 x-axis =0.3263, 0.2265, -0.9177), y-axis = (- 0.1652, -0.9423, -0.2912), z-axis = (-0.9307, 0.2466, -0.2701). Assignment of N2 x and y is arbitrary as this cluster is axial.

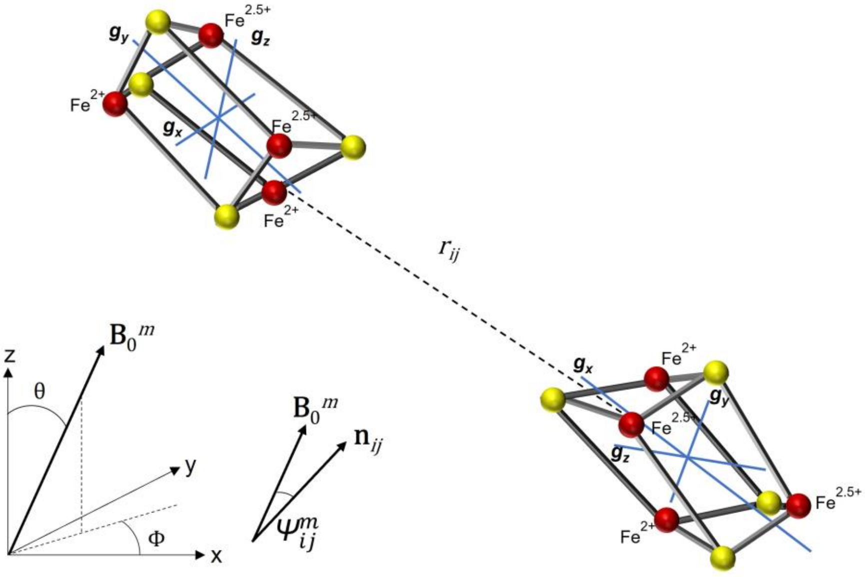

Supplementary Fig 7. EPR-based Potentiometric Redox Titrations of **a.** *T. elongatus* and **b.** *Synechocystis* PS-CI. The reduction potentials (mV vs SHE) of the samples are indicated on the right-hand side. Raw data in black and simulations in red. SDT = sodium dithionite.

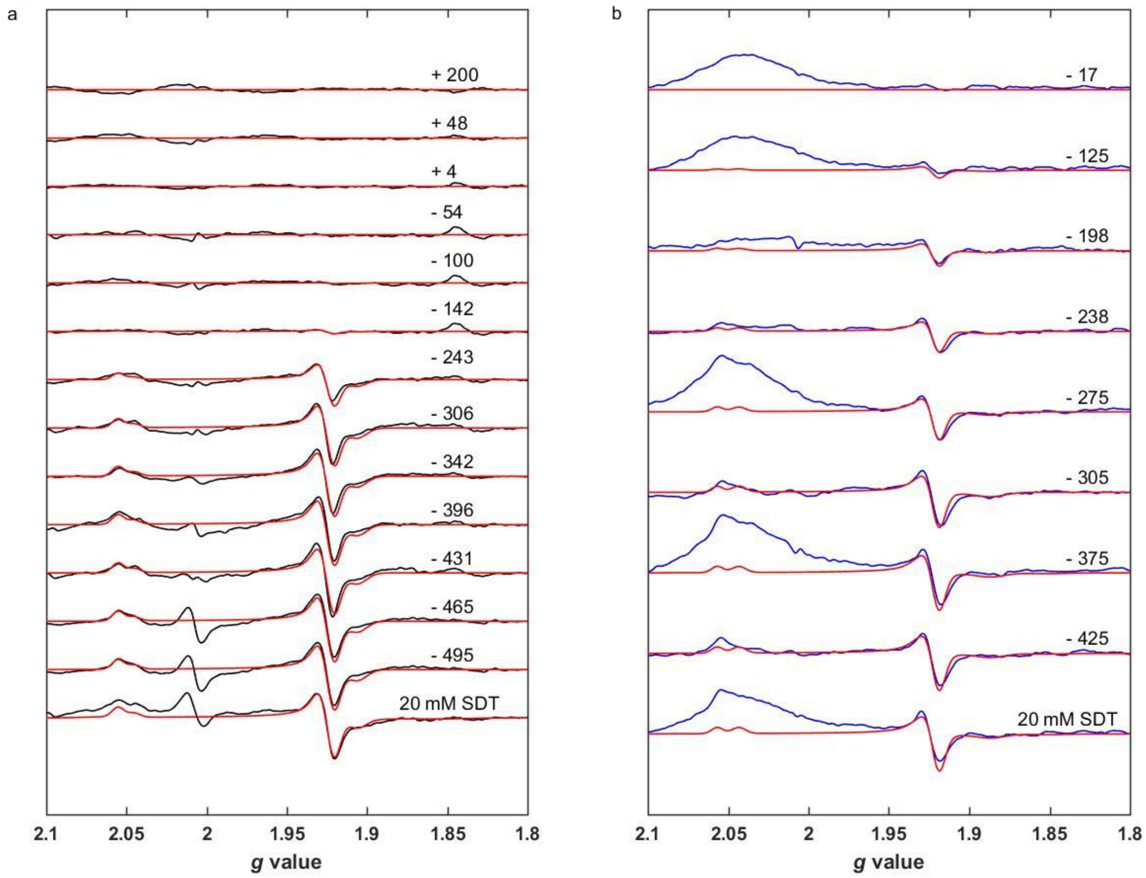

Supplementary Fig 8. Lack of N0 signal in *T. elongatus* PS-CI at −431 mV. EDFS spectra of *T. elongatus* titration sample at −431 mV measured at 10 K, 0.5 µs SRT, τ = 350 ns, π = 32 ns. N2 simulated in pink, N1 simulated in purple (1:1), sum of simulation in red, with no indication that N0 is reduced.

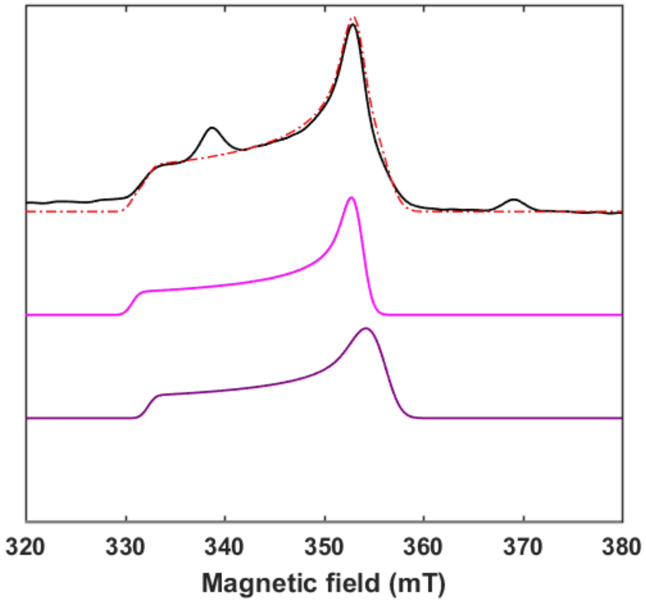

Supplementary Fig 9. Synthetic DNA sequence corresponding to the fragment of the *Synechocystis* sp. PCC6803 genome used to introduce a His-tag to the C-terminal end of the NDH-J protein.

GAAATGCCGGAGCCAAAGTATGTCATTGCCATGGGAGCCTGCACCATCACCGGTGGTATGTTTAGCAGTGATT
CCACCACAGCGGTAAGGGGGGTAGATAAATTGATTCCGGTGGATGTGTATATTCCCGGTTGCCCTCCCCGCCC
CGAAGCCATTTTTGATGCCATTATTAAACTGCGCAAAAAGGTGGCCAATGAATCTATCCAAGAACGGGCCATT
ACTCAGCAAACCCATCGCTACTACAGCACTTCCCACCAAATGAAAGTGGTGGCGCCGATTTTGGACGGCAAGT
ATTTACAACAGGGTACCCGTTCCGCTCCCCCCCGGGAGTTGCAGGAGGCCATGGGAATGCCTGTACCTCCCGC
TTTGACCACCTCTCAACAAAAGGAGCAATTAAACCGTGGCTGAGGAAGTGAACTCCCCCAATGAAGCTGTTAA
TCTCCAAGAGGAGACGGCGATCGCCCCTGTGGGCCCTGTATCCACCTGGCTGACCACCAATGGCTTTGAGCAC
CAGAGCTTAACGGCGGATCACCTGGGGGTGGAAATGGTGCAAGTGGAAGCGGATTTGCTTCTGCCCCTCTGT
ACGGCTTTATATGCCTATGGGTTCAACTATTTACAATGCCAGGGAGCCTACGATGAAGGGCCTGGTAAATCCT
TAGTGAGTTTTTACCATCTGGTGAAGTTGACCGAGGATACCCGTAACCCGGAAGAGGTCAGACTAAAAGTATT
TTTGCCCAGGGAAAATCCGGTGGTGCCCTCTGTGTACTGGATTTGGAAAGCGGCAGACTGGCAAGAGCGGGA
ATGCTACGACATGTTCGGCATTGTCTATGAAGGTCACCCCAATTTGAAACGGATTTTGATGCCGGAGGATTGG
GTAGGCTGGCCCCTGCGTAAGGATTATATTTCCCCCGATTTTTACGAACTCCAGGATGCCTATATCGAAGGTAG
GCATCATCATCATCATCATTAGTCCAGGCATCAAATAAAACGAAAGGCTCAGTCGAAAGACTGGGCCTTTCGT
TTTATCTGTTGTTTGTCGGTGAACGCTCTCTACTAGAGTCACACTGGCTCACCTTCGGGTGGGCCTTTCTGCGTT
TATACCCGGTCAGGCCTGCCCACGGGAGCTCCAAAATTAAACATCAAAATTAGTTTTAGCTCCCCCTTCCCCCA
CTGTTGCCAATGGACTGAAAACTTTGGGGGAATATTTTTGTGTTGACTGATTTTCAATCGGTTAAAACAACAGA
AAAAAAGGCTTTGATGGTGGCAAGAACGCTAAGGGAGGTAAGCCTTATGTCGTAGTTGCTTTTCAGGAGCTT
GTCTTGGGATTATTGGCAATGGAGGCCAATACTTCAGAGCGACTTGTGGAAATTAGCTACTAATTTATAGCTA
ATTTAATTAATTATGAGGCGTCTTACCTGGGTTTTGTGGTAATATATTGGAAGAATAACACAAGAATCCATAAA
CAACTAAATCAGTCATGGCTTACGATCTTGATTTACGTATTAAGGTAA

References

1. Maly, T. *et al.* Relaxation Filtered Hyperfine (REFINE) Spectroscopy: A Novel Tool for Studying Overlapping Biological Electron Paramagnetic Resonance Signals Applied to Mitochondrial Complex I. *Biochemistry* **43**, 3969–3978 (2004).
2. Eaton, S. S., Eaton, G. R. Relaxation times of organic radicals and transition metal ions. *Biol. Magn. Reson* **19**, 29–154 (2002).
3. Roessler, M. M. *et al.* Direct assignment of EPR spectra to structurally defined iron-sulfur clusters in complex I by double electron-electron resonance. *Proc. Natl. Acad. Sci. U. S. A.* **107**, 1930–1935 (2010).
4. Lovett, J.E., Bowen, A. M., Timmel, C.R., Jones, M.W., Dilworth, J.R., Caprotti, D., Bell, S. G., Wong, L.L., Harmer, J. Structural information from orientationally selective DEER spectroscopy. *Phys. Chem. Chem. Phys.* **11**, 6626–6637 (2009).
5. Bencini, A. & Gatteschi, D. Resonance of Exchange Coupled Systems. (1990).
6. Bertrand, P. *et al.* Biological polynuclear clusters coupled by magnetic interactions: From the point dipole approximation to a local spin model. *J. Am. Chem. Soc.* **116**, 3078–3086 (1994).
7. Elsässer, C., Brecht, M. & Bittl, R. Pulsed electron-electron double resonance on multinuclear metal clusters: Assignment of spin projection factors based on the dipolar interaction. *J. Am. Chem. Soc.* **124**, 12606–12611 (2002).
8. Elsaesser, C., Brecht, M. & Bittl, R. Treatment of spin-coupled metal-centres in pulsed electron – electron double-resonance experiments. 15–19 (2005).
9. Schuller, J. M. *et al.* Structural adaptations of photosynthetic complex I enable ferredoxin-dependent electron transfer. *Science* **260**, 257–260 (2019).
10. Djafarzadeh, R. *et al.* Biophysical and structural characterization of proton-translocating NADH-dehydrogenase (complex I) from the strictly aerobic yeast Yarrowia lipolytica. *Biochim. Biophys. Acta - Bioenerg.* **1459**, 230–238 (2000).
11. Narayanan, M. *et al.* Semiquinone and cluster N6 signals in his-tagged proton-translocating NADH:ubiquinone oxidoreductase (complex I) from Escherichia coli. *J. Biol. Chem.* **288**, 14310–14319 (2013).
12. Hinchliffe, P., Carroll, J. & Sazanov, L. A. Identification of a novel subunit of respiratory complex I from Thermus thermophilus. *Biochemistry* **45**, 4413–4420 (2006).
