# Supplementary material for: Functional basis of electron transport within photosynthetic complex I
Source: Nat Commun. 2021 Sep 10;12:5387. doi: 10.1038/s41467-021-25527-1 (PMC8433477; doi:10.1038/s41467-021-25527-1)
Supplement: Supplementary file 3 — Reporting Summary [file 41467_2021_25527_MOESM3_ESM.pdf]

nature portfolio

Corresponding author(s): GT Hanke, MM Roessler

Last updated by author(s): 26/07/21

Reporting Summary

Nature Portfolio wishes to improve the reproducibility of the work that we publish. This form provides structure for consistency and transparency in reporting. For further information on Nature Portfolio policies, see our Editorial Policies and the Editorial Policy Checklist.

Statistics

For all statistical analyses, confirm that the following items are present in the figure legend, table legend, main text, or Methods section.

n/a	Confirmed	
☐	☒	The exact sample size (*n*) for each experimental group/condition, given as a discrete number and unit of measurement
☐	☒	A statement on whether measurements were taken from distinct samples or whether the same sample was measured repeatedly
☒	☐	The statistical test(s) used AND whether they are one- or two-sided<br>*Only common tests should be described solely by name; describe more complex techniques in the Methods section.*
☒	☐	A description of all covariates tested
☒	☐	A description of any assumptions or corrections, such as tests of normality and adjustment for multiple comparisons
☒	☐	A full description of the statistical parameters including central tendency (e.g. means) or other basic estimates (e.g. regression coefficient) AND variation (e.g. standard deviation) or associated estimates of uncertainty (e.g. confidence intervals)
☒	☐	For null hypothesis testing, the test statistic (e.g. *F*, *t*, *r*) with confidence intervals, effect sizes, degrees of freedom and *P* value noted<br>*Give P values as exact values whenever suitable.*
☒	☐	For Bayesian analysis, information on the choice of priors and Markov chain Monte Carlo settings
☒	☐	For hierarchical and complex designs, identification of the appropriate level for tests and full reporting of outcomes
☒	☐	Estimates of effect sizes (e.g. Cohen's *d*, Pearson's *r*), indicating how they were calculated

Our web collection on statistics for biologists contains articles on many of the points above.

Software and code

Policy information about availability of computer code

Data collection	EPR data was collected using Xepr software (Version 2.1) commercially available from Bruker and installed on Bruker EPR spectrometers. Mass spectrometry data was collected using Q Exactive Plus Tune 2.3 commercially available from Oribtrap (part of Thermofisher).
Data analysis	Data was analysed in Matlab R2017b (version 9.3.0.713579) which is commercially available with the Curve Fitting Toolbox (Version 3.5.5). EPR data was analysed using the open source and freely available Easyspin programme (version 5.2.33). Peptide and protein identification were searched using MaxQuant (version 1.6.14.0).

For manuscripts utilizing custom algorithms or software that are central to the research but not yet described in published literature, software must be made available to editors and reviewers. We strongly encourage code deposition in a community repository (e.g. GitHub). See the Nature Portfolio guidelines for submitting code & software for further information.

Data

Policy information about availability of data

All manuscripts must include a data availability statement. This statement should provide the following information, where applicable:

- Accession codes, unique identifiers, or web links for publicly available datasets
- A description of any restrictions on data availability
- For clinical datasets or third party data, please ensure that the statement adheres to our policy

Source data are provided with this paper. The data generated in this study have been deposited in the Imperial College London Research Data Depository database under DOI: 10.14469/hpc/8656.
Accessions for proteins and protein subunits used in this work are as follows: Thermosynechococcus elongatus photosynthetic complex I, 6HUM (www.wwpdb.org/

pdb?id=pdb_00006hum) and NdhF1, Q8DKX9 (www.uniprot.org/uniprot/Q8DKX9); Synechocystis sp PCC 6803 Ndh-J, P19125 (www.uniprot.org/uniprot/P19125); Synechocystis sp. PCC6803 proteome (www.uniprot.org/proteomes/UP000001425).

Field-specific reporting

Please select the one below that is the best fit for your research. If you are not sure, read the appropriate sections before making your selection.

☒ Life sciences ☐ Behavioural & social sciences ☐ Ecological, evolutionary & environmental sciences

For a reference copy of the document with all sections, see nature.com/documents/nr-reporting-summary-flat.pdf

Life sciences study design

All studies must disclose on these points even when the disclosure is negative.

Sample size	No sample size calculation was performed. For EPR spectroscopy a sample size of one is sufficient as it contains a population of individual paramagnets representative of the protein sample.
Data exclusions	No data was excluded from the analysis.
Replication	The EPR data have been replicated and assessed under different condition (as detailed in the manuscript) on 3 independent spectrometers with 3 batches of protein used in the study.<br>EPR is based on non-destructive absorption of electromagnetic radiation by the sample. The measurable quantity is reflective of a population distribution across many individual paramagnetic within each sample. It is therefore not normally necessary to repeat EPR measurement multiple times or on different spectrometers. Nonetheless, for logistical reasons, in this study three different EPR spectrometers were used.<br><br>Mass spectrometry was performed once for each complex purified from T.elongatus and Synechocystis to confirm the presence of the appropriate subunits. Replication was not necessary as no quantitative comparisons were made between experiments.
Randomization	Randomization and covariates are not applicable to this study as it comprises experiments on two purified proteins of known origin. The proteins were purified from multiple cyanobacterial cultures grown in controlled conditions, where little or no physiological variation is expected to occur.
Blinding	Blinding was not applicable to the study as it did not involve comparison between samples or populations. Experiments were carried out on two purified, known protein complexes.

Reporting for specific materials, systems and methods

We require information from authors about some types of materials, experimental systems and methods used in many studies. Here, indicate whether each material, system or method listed is relevant to your study. If you are not sure if a list item applies to your research, read the appropriate section before selecting a response.

Materials & experimental systems

n/a	Involved in the study	
☒	☐	Antibodies
☒	☐	Eukaryotic cell lines
☒	☐	Palaeontology and archaeology
☒	☐	Animals and other organisms
☒	☐	Human research participants
☒	☐	Clinical data
☒	☐	Dual use research of concern

Methods

n/a	Involved in the study	
☒	☐	ChIP-seq
☒	☐	Flow cytometry
☒	☐	MRI-based neuroimaging
